# Real-Time Social Data Collection in Rural Bangladesh via a ‘Microtasks for Micropayments’ Platform on Android Smartphones

**DOI:** 10.1371/journal.pone.0165924

**Published:** 2016-11-10

**Authors:** Andrew Reid Bell, Patrick S. Ward, Mary E. Killilea, Md. Ehsanul Haque Tamal

**Affiliations:** 1 Department of Environmental Studies, New York University, 285 Mercer St., New York, NY, 10003, United States of America; 2 International Food Policy Research Institute (IFPRI), 2033 K St. NW, Washington, DC, 20006, United States of America; 3 International Food Policy Research Institute (IFPRI) Dhaka Office, House 10A, Road 35, Gulshan 2, Dhaka, 1212, Bangladesh; Institut Pasteur, FRANCE

## Abstract

The advent of cheap smartphones in rural areas across the globe presents an opportunity to change the mode with which researchers engage hard-to-reach populations. In particular, smartphones allow researchers to connect with respondents more frequently than standard household surveys, opening a new window into important short-term variability in key measures of household and community wellbeing. In this paper, we present early results from a pilot study in rural Bangladesh using a ‘microtasks for micropayments’ model to collect a range of community and household living standards data using Android smartphones. We find that more frequent task repetition with shorter recall periods leads to more inclusive reporting, improved capture of intra-seasonal variability, and earlier signals of events such as illness. Payments in the form of mobile talk time and data provide a positive development externality in the form of expanded access to mobile internet and social networks. Taken to scale, programs such as this have potential to transform data collection in rural areas, providing near-real-time windows into the development of markets, the spread of illnesses, or the diffusion of ideas and innovations.

## Introduction

How much did you spend on food last year? How many days did you miss work last summer?

These would be difficult questions for most people to answer with any precision. However, the structure of most household surveys–with return visits often years apart, and recall periods (the length of time over which a respondent is asked to recall something like spending, labor, or consumption) of months or longer–requires participants to do so. Do these data really provide meaningful signals or characterizations upon which to base development policies and interventions? The advent of smartphones into these same areas presents a new opportunity to engage rural peoples, and researchers are beginning to explore using smartphones to gather information for particular issues such as food prices [1], food security [2], or disease symptoms [3,4]. In this paper, we present early results from a study of smartphone-based data collection that show different response patterns when respondents are given tasks more frequently or with shorter recall periods. Taking these results to scale and embracing smartphones as a medium of exchange could completely restructure the connection that researchers, policymakers, and development practitioners share with subjects in rural communities.

Within the international development and research communities, household surveys have long been the principal building block used to measure and monitor living standards (e.g., [4,5]), assess impacts of development strategies and policy reforms (e.g., [6,7]), and test theories about household behavior (e.g., [8,9]). Capturing representative signals from rural populations over time, however, is typically a logistical challenge. The population of interest may be difficult or expensive to reach, and therefore survey rounds are often restricted to no more than a few return visits in the timeframe of a typical research project, with many occurring only once. During these visits, investigators engage respondents in cognitively taxing exercises for an extended period during which respondents may grow fatigued or may be drawn away by other obligations, potentially resulting in less reliable responses. Additionally, given the infrequency of data collection efforts, recall periods may be inappropriately long or miss much of the intervening time between survey waves, potentially ignoring important sources of exogenous variation. Data relevant for risk assessment and modeling (such as recall of extreme events, illnesses, or accounting for labor or other market opportunities) can exhibit a great deal of temporal variability, and are difficult to capture reliably. A mismatch across the scales at which change occurs, at which respondents can recall them, and at which investigators are able to reach and interview them thus prevents the development community from meaningfully capturing the complexity of behavioral responses to environment and other constraints.

It is reasonable to expect smartphone penetration into rural areas will follow the path taken by mobile phones, extending beyond the young and wealthy, to reach most households within a few years [10]. In Bangladesh, smart phone use nearly tripled over the year 2015 to over 8 million users [11]. While much of the current development in information and communication technologies in rural areas is focused on SMS text, voice-based programs, and the potential for mobile phones to deliver information, our hopes rest in the long-run potential of smartphones to engage rural populations more deeply in a mutual exchange of information. Other research groups have begun to explore the potential of Android mobile devices for data collection in recent years, with notable examples including the EpiCollect platform [12] and the SATIDA COLLECT [2] platform, both leveraging the capabilities of Android devices (with cameras, location sensors, etc.) to enable trained surveyors to capture a range of social and natural systems data. However, while these and other authors [13–15] acknowledge the potential for “citizen science” on these platforms, with respondents engaging directly with the data collection platform in the absence of trained professionals, we have not found examples in the literature that describe participants’ ability or willingness to do so, particularly in hard-to-reach, rural areas. This is perhaps simply because the opportunity itself is relatively new, with smartphones not yet as widely used as mobile phones, and we aim to begin filling this gap with the current study.

We present some early highlights from a study of smartphone-based data collection in rural Bangladesh that demonstrate the benefits of regular engagement with rural respondents via smartphone, and that highlight key areas for calibrating participation in a scaled up program. Specifically, we show in the results and analysis that follows that households report significantly more income-generating activity when asked to recall activities on a weekly (as opposed to monthly, or seasonal) basis. We also note that even when recall periods are held constant (e.g. what was your access to clean drinking water over the last 7 days?), results vary depending on the frequency of data collection, highlighting the temporal variability that is simply missed by survey waves that are spaced months or years apart. Importantly, we also demonstrate the capacity for signals of public health (such as school days missed due to illness in a region) to appear significantly earlier when survey frequency is higher–of great value to the development of tools to track diffusion of disease, technology, or ideas across space and time.

## Materials and Methods

The study on which we report uses a customized interface for Open Data Kit (ODK) [16] on Android smartphones to interact with participants on a ‘micro-task for micro-payment’ [17] basis in rural Rangpur division in northwestern Bangladesh. Participants in our study have the opportunity to respond to short survey tasks (each requiring roughly 3–10 minutes) in return for small payments in the form of pre-paid mobile data, SMS messaging, and talk time. Each week, several different survey tasks can be ‘pushed’ to each handset, with tasks varying in value on a scale from 1 to 5, with each increment worth an additional 10MB in data and 5 Taka (about 0.06 USD) in talk time and/or SMS messaging. Task value was determined by the research team a priori and was a combined function of expected cognitive and temporal burdens, as well as the perceived research value of information. Though each task is small in scope, in content they are consistent with standards of traditional household surveys. Tasks are structured and valued so that active engagement with the program can potentially pay for all of the user’s mobile talk, text, and data needs–a positive externality of expanding access to information via the internet. Some tasks–such as diaries of food consumption, freshwater access, instances of sickness or school absence, and participation in wage labor–are repeated regularly, so that intra-annual or even intra-seasonal variability are revealed in the data. We prepared several different versions of each task, so that some participants may be asked to report on food or fertilizer application on a weekly basis, while others only once a month, and still others only once a season (three months). Some participants may be asked to report on their own behavior and experiences, while others may be tasked with ‘crowdsourcing’ data collection–engaging friends, neighbors, etc.–to report the same information. The rewards for different tasks vary (based on perceived task difficulty or anticipated time commitment), with the mix of weekly, monthly, and seasonal tasks sent to each phone randomized and balanced to standardize potential earnings from continued engagement throughout the project’s duration, funded at the time of writing to continue through 50 weeks of data collection.

### Sample selection

**Fig 1:**
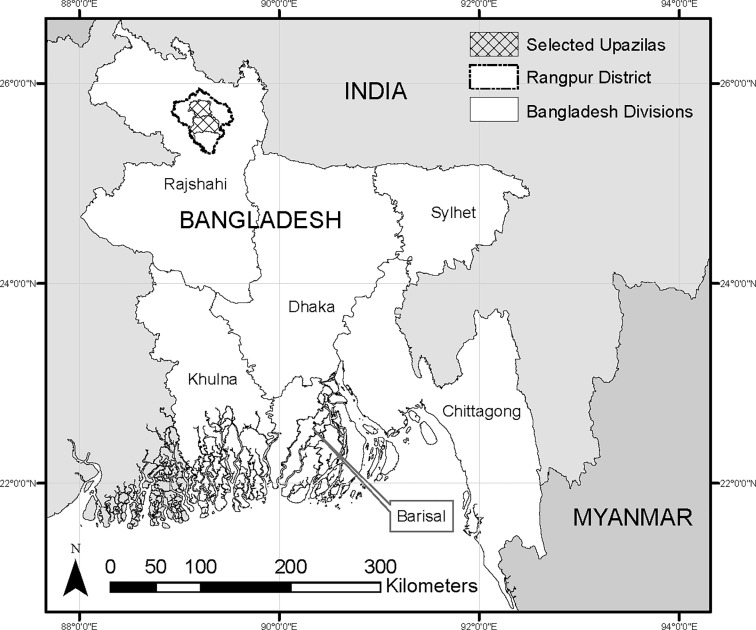
Study Area.

**Fig 2:**
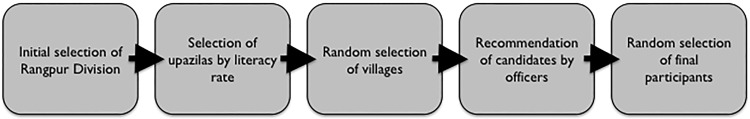
Sample selection pathway.

The central design criterion underlying our sample selection was to capture ‘potential early adopters,’ people in rural Bangladesh who might reasonably be expected to be among the first to obtain smartphones. We focused our case study in Rangpur district, a rural district in Rangpur division in northwestern Bangladesh (Fig 1). From the eight upazilas (sub-district administrative units) in the district, we selected the two with the highest literacy rates in the 2011 Bangladesh census [18]–Mithapukur and Rangpur Saddar. We randomly selected 40 villages from a pooled list of all villages in the two upazilas, and for each selected village, solicited a short list of 25 potential participants from the local agricultural extension officer. The officer was asked to recommend farmers with whom they had had contact, who were known to have or use mobile phones, and who might be inclined to use a smartphone. This approach focuses only on officers’ assessments of technical capacity, and does not explicitly target gender or any social class. From this list of 25 names provided, we randomly selected 12–13 participants for our study (for a total of 480 participants across the 40 villages). The different selection processes in each stage are summarized in Fig 2. By soliciting a larger number of names directly and then randomly selecting a subset, we hoped to better capture aptitude for smartphones than would be possible in a simple random survey, but avoid any issues of patronage that could arise through direct solicitation of names. Basic demographic characteristics of our sample are provided in Table 1, compared against two other benchmarks: a representative sample of household heads in Rangpur Division (from 2011), and the Bangladesh average (from 2015). As might be expected from our design and sample eligibility criteria, our sample is typically younger, more literate, and more highly educated than a typical household head in roughly the same area. However, like the Rangpur sample, our sample is much more heavily male than the true sex ratio of the country.

**Table 1:** Sample characteristics, compared against a representative Rangpur Sample, and the Bangladesh average.

	(1)	(2)	(3)
Demographic variable	This Study (2016)	Rangpur Division (household-head) (2011) [19]	Bangladesh average (2015) [20]
Average Age	32.9 (11.8)	44.2 (13.79)	26.3*
Sex Ratio (Male to Female)	8.53	10.44	0.91
Average Years of Education	9.88 (3.71)	3.45 (4.42)	10
Fraction of sample identifying as literate (able to read and write)	0.89	0.45	0.615

*Median age.

Standard deviations in parentheses.

Selected participants in all villages attended a full-day training to sensitize them on the use of the smartphone (including ancillary–albeit important–features of the handset, such as how to make calls, how to send SMS messages, how to take and access photographs, etc.), the use of the customized ODK interface, the structure of our pilot, and finally to give them the opportunity to practice completing several different tasks in a controlled environment. Participants were then issued a smartphone for their use during the study. All participants were issued the same device, a Symphony Roar V25 device using Android 4.4.2, as set up for sale by Banglalink. While devices were initially procured and distributed by the research team, study participants had the opportunity to earn enough ‘credits’ (through continued engagement with the study) to keep the handset upon the culmination of the study. Written consent was obtained from participants at the time of training; this study and consent procedure was approved by the IFPRI IRB (IRB #00007490, FWA #00005121) on September 21, 2015. The IRB application number is 2015-49-EPTD-M.

### Experimental design—tasks and platform

**Fig 3:**
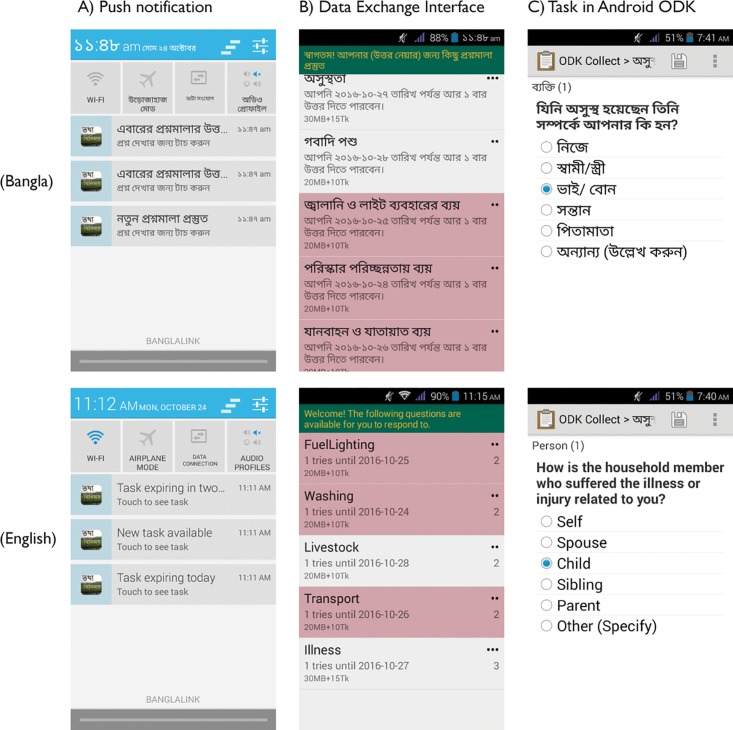
Custom interface presents tasks to participants. Selecting an available task (at left) launches the task in ODK (at right).

Participants were given the opportunity to complete tasks coded in ODK [16,21], via a custom interface developed by Nafundi (the developers of ODK; https://nafundi.com/) that presented respondents with a list of tasks currently available to them, the validity period of the tasks, and their values (Fig 3). ODK is an open-source survey platform designed as a local application that can be installed on mobile devices running the Android operating system. ODK is widely used in field research and data collection, as it allows researchers to design surveys that enable responses to survey tasks (coded to include standard data collection inputs such as open text inputs, check boxes, dropdown menus, as well as smartphone-specific tools such as images, locations, and free-form sketches) with finger taps and swipes [16]. Our customized interface, named “Data Exchange,” alerted participants when new tasks were available or were close to expiring via push notifications (messages sent from an application to a device’s status bar); selecting a task from the “Data Exchange” interface launched the task in the ODK platform. Completed tasks are sent to a central server at whatever point in time the device is connected to a mobile data network or Wi-Fi.

We prepared a total of 46 different survey tasks that varied in difficulty, typically requiring from 3 to 10 minutes to complete. Tasks were assigned a value ranging from 1 to 5 points, indicating the reward for completion, with a value of 1 corresponding to 10MB of data and 5 Taka of talk time and 5 corresponding to 50MB of data and 25 Taka of talk time. Each task’s value was determined by the research team a priori and was a combined function of expected cognitive and temporal burdens, as well as the perceived research value of information.

We prepared different versions of each of these 46 tasks, varying (i) the frequency (week, month, season) with which the task would re-occur (and in many cases, the period over which the task would require participants to recall), and (ii) whether the task was to be completed from the participant’s own recollection or if it was to be ‘crowdsourced.’ Crowdsourced tasks required participants to first complete the task from their own recollection as before, but then to select a new person (a friend, neighbor, or even a stranger) and solicit responses to the task, as though conducting a survey. The complete list of tasks, as well as the versions of each task that were prepared, is in S1 Table.

We created 20 unique smartphone setups, with each of our 480 participants randomly assigned to one of the 20 setups (for a total of 24 participants with each setup). Each setup included exactly one version of each of the 46 tasks (e.g., Task 12, repeated monthly, not crowdsourced). Since a task repeated weekly would be worth much more over the duration of the pilot than the same task repeated only monthly or seasonally, the distribution of task versions across setups needed to be carefully calibrated to achieve parity in earnings potential (i.e., the total value of talk-time and data re-charges a participant could earn over the duration of the study by completing all tasks). We achieved parity across smartphone setups by first randomly assigning one version of each task to each setup, and then (via a Matlab script) making pairwise switches of task versions between smartphone setups until the Gini coefficient of earnings potential across the phones fell below 0.001. The Gini coefficient is a common measure of the inequality of a distribution, with coefficients ranging from 0 (value spread equally across members) to 1 (value completely centralized in one member), computed as

$$G=\frac{2}{n}∙\frac{\sum_{i}iy_{i}}{\sum_{i}y_{i}}-\frac{n+1}{n}$$

where *y*_*i*_ are the *n* members of the set, with values ordered from least (*i* = 1) to greatest (*i* = *n*), such that *y*_*i*_ is less than or equal to *y*_*i* + 1_.

### Payments

The calibrated smartphone setups provided respondents with 5–10 tasks per week, with an average total value of 30 points (300MB + 150 Taka talk-time) per week if all tasks were completed. All tasks completed by respondents were received in our ODK Aggregate server, which were downloaded daily. Data were stored for subsequent processing and analysis, while a list of completed tasks, task values, and SIM card identifiers were uploaded to a specialized portal created by our mobile partner Banglalink, who then recharged participant SIM cards the following day. Additionally, participants were eligible to ‘earn’ their smartphone (i.e., retain the handset after the termination of the study period) by obtaining at least 400 points across the duration of the pilot study (possible in as little as 13–14 weeks with full participation).

## Results and Discussion

### Sample results from changing recall periods

Our results suggest that participants that are asked to recall recent activities respond differently than participants with longer recall periods. For example, our results demonstrate that, on average, those tasked with reporting income-generating activities once per season (125 days)–reporting for the whole season–list activities summing to about 9 person-hours per day across the entire household. For those asked to respond once a month–for a one-month recall–this value more than doubles, to an average of 22 person-hours per day. Those tasked with tallying their activities weekly report more than 50 person-hours per day engaged in income generation of some form (Table 2). Additionally, the average earnings per person-hour across these different recall periods differ by an order of magnitude (with average wage rates lower for shorter recall periods), and help to explain the gulf in reported activities: at the end of a season, the higher-earning activities remain salient enough to report, while the lower-earning activities are less likely to be reported.

**Table 2:** Selected differences across task frequency.

**Income variables**	**Task Frequency (in bold letters)**					
	(Recall period in parentheses)					
	**Season**		**Month**		**Week**	
	(Season)		(Month)		(Week)	
Household daily labor hours (per day-household)*	9.1393	M,W	22.0649	S,W	53.6065	S,M
Household wage rate (Taka per hour)*	40.6006	W	17.1266	W	9.6422	S,M
**Climate event expectation variables**	**Season**		**Month**			
	(Season) (84 Respondents)		(Month) (265 Respondents)			
Expected risk of flood occurrence during recall period (Scale 1 to 5)*	1.7159	M	1.2066	S		
Expected flood damage over recall period (Scale 1 to 5)*	1.875	-	1.8212	-		
Expected risk of drought occurrence during recall period (Scale 1 to 5)*	2.8295	-	2.5972	-		
Expected drought damage over recall period (Scale 1 to 5)*	2.375	M	2.1337	S		
Expected risk of cyclone occurrence during recall period (Scale 1 to 5)*	2	M	1.2552	S		
Expected cyclone damage over recall period (Scale 1 to 5)*	2.3068	M	1.9253	S		
**Climate event experience variables**	**Season**		**Month**			
	(Season) (84 Respondents)		(Month, summed over all months in season) (265 Respondents)			
Fraction of participants reporting flood over previous season	0.2738	M	0.0943	S		
Fraction of participants reporting drought over previous season	0.5595	M	0.7698	S		
Fraction of participants reporting cyclone over previous season	0	M	0.0453	S		
**Water quality variables**	**Season**		**Month**		**Week**	
	(Week) (137 Respondents)		(Week) (196 Respondents)		(Week) (83 Respondents)	
Variation in reported water quality along study period (Variance of Scale 1 to 5)*	0.0398	M,W	0.3372	S,W	0.4589	S,M

S: Significantly different from Season result; M: Significantly different from Month result; W: Significantly different from Week Result (Using Chi-Square test (proportions) or Kolmogorov-Smirnov test (means) at 95%).

*Averaged across respondents.

Based on 13 weeks of data.

Similarly, the recall period shapes how participants report weather events and their concerns about them. On average, participants report higher average expectation of floods, droughts, and cyclones occurring (and with higher potential damages) over a season than over any given single month. This is not particularly surprising–hazards have greater opportunity to return and cause damage over longer periods than shorter periods. However, reporting of actual events experienced is very different. When recalling the previous season of realized weather, 27 percent of participants recall floods and 56 percent recall droughts, with no one recalling cyclones over the prior season. In contrast, only 9 percent of participants asked to recall on a monthly basis ever recall experiencing floods (with their monthly recall covering the same period of time), while 77 percent recall droughts and 5 percent report experiencing cyclones. What appears to be happening is that whether an event qualifies as an extreme and remains salient for reporting depends strongly on timeframe over which participants are asked to recall events occurring. While these assessments are purely subjective, these data suggest that while hazards leave real and immediate impacts on individuals’ perceptions, these perceived impacts are evidently smoothed over time. In the present pilot study, we lack a reliable, ground-truthed measure of the occurrence of these events against which to compare our observations and better distinguish the character of events that fade in memory from those that do not. The stark differences across recall periods highlight the importance of such ground-truthing in future work of this nature.

### Sample results from changing task frequency

Even when recall length is kept constant, asking at different frequencies reveals different patterns in response. In one task, participants were asked to report on their access to drinking water over the previous 7-day period, and to rate the quality of that drinking water on a scale of 1 to 5. Participants were randomized to receive this task once in 7 days, once in 30 days, or once during the course of a season, but in each case the recall period remained at 7 days. On average, the longitudinal variance (i.e., spread of an individual’s responses) on the quality of their drinking water differed significantly depending on how frequently they were asked. In this particular case, variance increased with frequency, capturing higher frequency variation in water quality that would otherwise not be captured. However, we are cautious with interpreting this finding, as it plausibly occurs in the opposite manner: a lower longitudinal variance as additional sampling over time reduces the importance of an outlying report of water quality. In either case, higher reporting frequencies improve the value of such diaries–whether of food, water, illness or other–by making the results less of an artifact of the particular week in which the survey was conducted.

**Fig 4:**
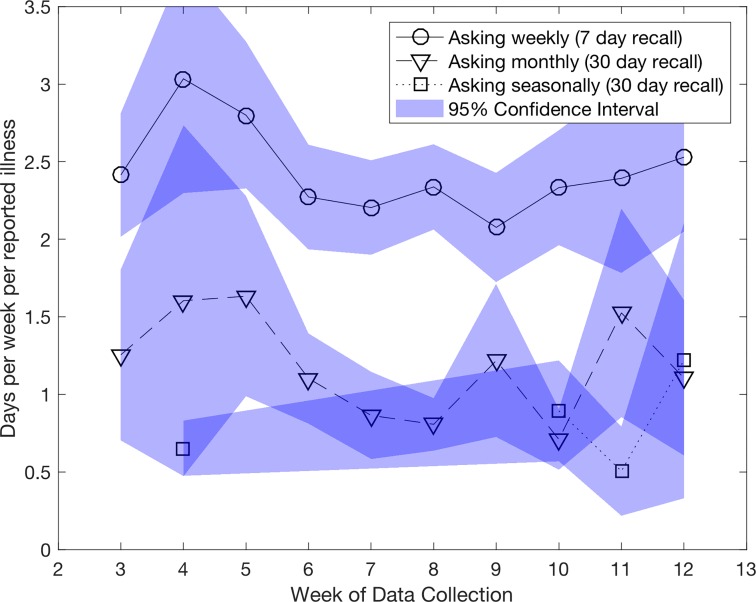
Average reported length of school absence due to illness in households.

Additionally, asking questions with higher frequencies can yield important information more rapidly. This is perhaps the most intuitive and most important result to highlight, and is likely to be of greatest value to policymakers and development practitioners who will have access to near-real-time characterizations of ground realities. For example, when asked about school attendance for their dependents, households reporting with a weekly recall report durations in illness nearly twice as long as those reporting on a monthly basis (as in our first result above) (Fig 4), but importantly, peaks in the length of absence in the weekly reporting precede those in monthly reports by at least one week (significant with 1 percent probability of Type I error by the Granger causality test). Being able to detect anomalies like this earlier rather than later could be of immense benefit to health and planning professionals, extending the reach of potential tracking tools like Google Trends [22] beyond urban areas and regular internet users. Consider the case of the 2014 Ebola outbreak in West Africa, where poor identification and reporting from rural areas contributed to the momentum of the disease outbreak [23,24], and the role that regular, statistically testable data exchange with such areas could play in managing and containing such challenges in future.

### Managing participation

**Fig 5:**
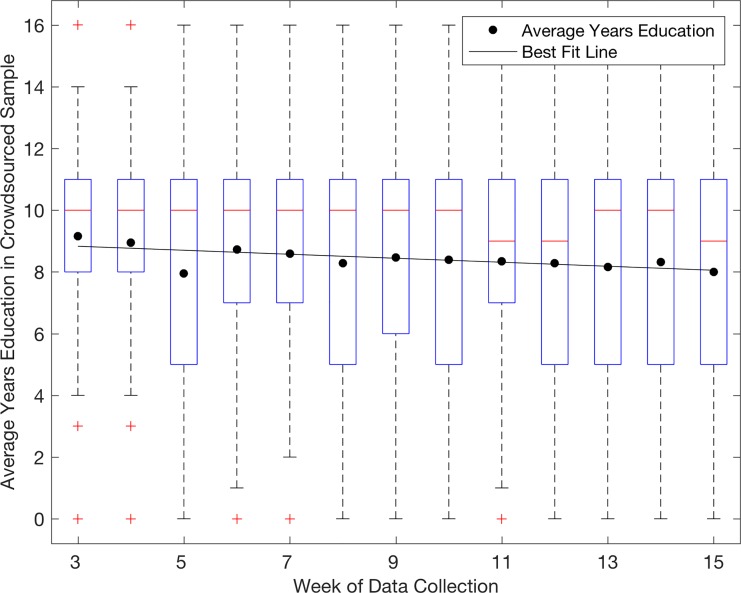
Box-whisker plot showing quartile distribution of years of education by week in the crowdsourced sample, as well as weekly average and best-fit (Tobit) regression, through week 15 of data collection. Best fit line from Tobit regression has a slope of -0.078 (years education per week), significant at p = 0.001.

Our study is only an early lens into the potential use of smartphones as a medium of exchange with rural populations. The extent to which participants’ signals reliably characterize the landscape in different contexts necessitates careful groundtruthing in each new domain. Our study embeds some clues as to what kinds of tools we have to address some issues of data reliability, namely sampling bias and response rates. One of the most pressing concerns in the near term is that smartphone adoption will likely be among the relatively young and technology-savvy, and this is reflected in our sample: participants in our sample are 33 years of age on average with 10 years of schooling, compared to an average of 44 years of age and 3.5 years of education among household heads across Rangpur division in a 2011 representative household survey [19]. Crowdsourcing provides a potential mechanism to overcome some of this bias: when our participants were tasked with collecting information on subjective well-being from others, they tapped into a sample of a similar age but significantly lower education (8.5 years)–a measurable step closer to the 3.5 years observed in the representative survey. Further, this average appears to be gradually converging on the representative average over time, perhaps as participants step further out of their more proximate social networks and capture a broader characterization of those around them (Fig 5). Promisingly, gender balance is significantly improved in the crowdsourced sample (27% female, compared with only 11% in our main sample of participants). This is still quite different from national sex ratios (approximately 52% of adults aged 25–54 are female [20]), but is a marked increment in that direction.

**Fig 6:**
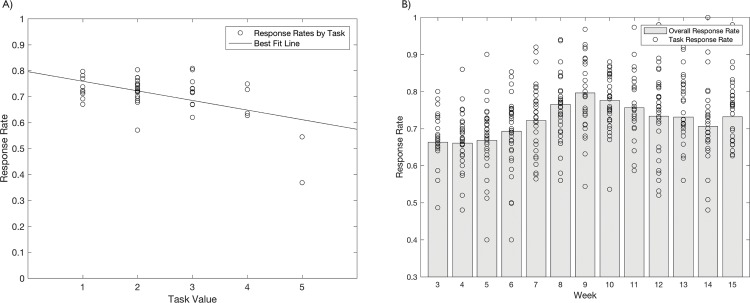
Response rate to tasks as function of a) task value and b) week. Tasks are valued on a scale of 1 to 5, with each increment worth an additional 10MB of data and 5 Taka (0.06 USD) in talk time.

Looking to response rates, we note that they are weakly negatively correlated with the value of the task completed, which is itself a function of the perceived complexity or estimated time commitment of the survey task (Fig 6A). It seems clear that the less our tasks threaten to occupy the participant for long durations (as would traditional household surveys), and the more they can meaningfully be split into smaller sub-tasks across the day or week, the higher the response rate and the stronger the link between researcher and respondent. A second important message is that, with response rates above 80% for our lowest-valued tasks (providing 10MB of data and the equivalent of about USD 0.06 of talk time), the marginal cost of a few minutes of a respondent’s attention is comparatively low. With a carefully structured task, researchers or policymakers could potentially observe a year’s worth of experiences related to food or work or emotional well-being, reported weekly, acquired for only a few US dollars.

## Conclusions

In this paper we have presented preliminary results from a pilot program in Bangladesh using a ‘microtasks for micropayment’ model to directly engage rural households in regular updates on their lives and communities. Taken to scale in a world in which smartphones have permeated remote rural areas, a program such as this could allow researchers across the world to test and deploy a range of survey and experimental tasks for a fee, with those proceeds translating into mobile data and talk-time access for the rural poor (and not into fuel costs to transport researchers and enumerators across the world, outside of periodic groundtruthing exercises). In place across the developing world, smartphone-based research programs could map out diffusion of new practice and technology as well as diagnose and track the spread of illnesses. Much as Amazon’s Mechanical Turk has democratized a wide range of game, choice, and other tasks [25], so the approach presented here extends our reach to more-distant populations and our ability to collect data and inform models of decision-making and system behavior in the developing world.

In this, we are taking a tool used already by market researchers in urban areas across the globe, and stepping forward to see how it could be applied in rural contexts in developing countries. Our study demonstrates that it has the potential to channel research resources more efficiently than a traditional household survey, and capture signals a traditional household survey never would.

The opportunity cost in this approach, of course, is loss of accountability and representativeness. This platform moves data collection from trained professionals and statistically designed samples to self-selected samples of non-representative participants whose abilities and goals will vary widely. New burdens on researchers for improving data quality will include making survey tasks intuitive, aligning rewards with activities so that participants earn the most by responding truthfully and carefully, and finding opportunities to overcome sample biases. Some of these challenges have analogs in traditional approaches–respondents tire, or choose not to respond truthfully–but in the mobile approach, with frequent engagement, there may be greater opportunity to detect and isolate such anomalies than in traditional survey waves. The question of bias will likely diminish as smartphone penetration into rural areas increases, and we hope to use the intervening period to develop a robust platform for high-resolution, near-real-time data collection that could help map the diffusion of new technologies, new diseases, new pests, and the impacts of climate shocks as they happen.

## Supporting Information

S1 Table Task list and versioning. (DOCX)
