# Supplementary material for: Real-Time Social Data Collection in Rural Bangladesh via a ‘Microtasks for Micropayments’ Platform on Android Smartphones
Source: PLoS One. 2016 Nov 10;11(11):e0165924. doi: 10.1371/journal.pone.0165924 (PMC5104491; doi:10.1371/journal.pone.0165924)
Supplement: S1 Table — (DOCX) [file pone.0165924.s001.docx]

**S1 Table: Task list and versioning**

| **Title** | **Once only, start of pilot** | **Once only, any time during pilot** | **Once per season** | **Once per month** | **Once per week** | **Crowd-sourced** | **Value** |
| --- | --- | --- | --- | --- | --- | --- | --- |
| Agricultural Extension Services | No | No | Yes | Yes | No | No | 1 |
| Agricultural Production | No | No | Yes | Yes | No | No | 3 |
| Agricultural Subsidy Card | No | No | Yes | No | No | No | 1 |
| Bad Shocks | No | No | Yes | Yes | No | No | 1 |
| Basic Information | Yes | No | No | No | No | No | 3 |
| Climate Event | No | No | Yes | Yes | No | No | 2 |
| Crops | No | No | Yes | No | No | No | 4 |
| Current Migrants | No | No | Yes | No | No | No | 2 |
| Drinking Water Diary | No | No | Yes | Yes | Yes | Yes | 4 |
| Employment | No | No | Yes | Yes | Yes | Yes | 3 |
| Facilities | Yes | No | No | No | No | No | 2 |
| Farm Labor | No | No | Yes | Yes | Yes | No | 3 |
| Fertilizers | No | No | Yes | Yes | No | No | 3 |
| Fish Pond Inputs | No | No | Yes | Yes | No | No | 2 |
| Fish Pond Production | No | No | Yes | Yes | No | No | 3 |
| Food Consumption | No | No | Yes | Yes | Yes | Yes | 4 |
| Food Storage Capacity | No | No | No | Yes | Yes | No | 2 |
| Good Shocks | No | No | Yes | Yes | No | No | 1 |
| Household Composition | Yes | No | No | No | No | No | 3 |
| Housing and Sanitation | Yes | No | No | No | No | No | 2 |
| Illness | No | No | Yes | Yes | Yes | Yes | 3 |
| InformationTools | No | No | Yes | Yes | No | Yes | 2 |
| Internet Use | No | No | No | Yes | No | No | 2 |
| Irrigation | No | No | Yes | Yes | No | No | 3 |
| Latrine Use | Yes | No | No | No | No | No | 2 |
| Latrine Walk | No | Yes | No | No | No | No | 5 |
| Livestock | No | No | Yes | Yes | No | No | 2 |
| Loans | No | No | Yes | Yes | No | No | 1 |
| Marketing | No | No | Yes | Yes | Yes | No | 3 |
| Non Agricultural Enterprise | Yes | No | No | No | No | No | 1 |
| Non Food Expenditure Cosmetics | No | No | Yes | Yes | Yes | No | 2 |
| Non Food Expenditure Fuel Lighting | No | No | Yes | Yes | Yes | No | 2 |
| Non Food Expenditure Transport | No | No | Yes | Yes | Yes | No | 2 |
| Non Food Expenditure Washing | No | No | Yes | Yes | Yes | No | 2 |
| Other Income | No | No | Yes | Yes | No | No | 1 |
| Plots | Yes | No | No | No | No | No | 4 |
| Remittance In | No | No | Yes | Yes | No | No | 2 |
| Remittance Out | No | No | Yes | Yes | No | No | 2 |
| Rental Tools | No | No | Yes | No | No | No | 2 |
| Safety Programs | No | No | Yes | No | No | No | 1 |
| Savings | No | No | Yes | Yes | No | No | 1 |
| School Attendance | No | No | Yes | Yes | Yes | Yes | 2 |
| Subjective Wellbeing | No | No | Yes | Yes | Yes | Yes | 3 |
| Transportation Tools | No | No | Yes | Yes | No | Yes | 2 |
| Tubewell | No | Yes | No | No | No | No | 5 |
| Work Animals | No | No | Yes | Yes | No | No | 2 |
